# Comparison on the reflux and nutritional status of different reconstruction methods after laparoscopic proximal gastrectomy: a systematic review and network meta-analysis

**DOI:** 10.1007/s13304-025-02324-9

**Published:** 2025-11-06

**Authors:** Chenglin Xin, Zimeng Wang, Zhi Zheng, Shitan Lu, Xicheng Wei, Jun Zhang, Jie Yin, Zhongtao Zhang

**Affiliations:** 1 Department of General Surgery, Beijing Friendship Hospital, Capital Medical University; National Clinical Research Center for Digestive Diseases, 95 Yong-an Road, Xi-Cheng District, Beijing, 100050 China; 2 State Key Lab of Digestive Health, Beijing, 100050 China; 3 Beijing Key Laboratory of Cancer Invasion and Metastasis Research, Beijing, 100050 China

**Keywords:** Proximal gastric cancer, Laparoscopic proximal gastrectomy, Digestive tract reconstruction, Postoperative reflux, Nutritional status, Network meta-analysis

## Abstract

**Background:**

The rising prevalence of gastric cancer in the upper third of the stomach has generated considerable interest in laparoscopic proximal gastrectomy (LPG). Traditional esophagogastric anastomosis after LPG has been associated with postoperative reflux issues. Despite the availability of various improved reconstruction techniques, there is still ongoing debate on the optimal approach. This network meta-analysis seeks to assess the reflux and nutritional outcomes associated with various reconstruction techniques subsequent to LPG.

**Methods:**

A comprehensive literature search was performed across five databases: PubMed, Medline, Embase, Cochrane Library, and Web of Science. The reconstruction methods following LPG include esophagogastrostomy (EG), jejunal interposition (JI), jejunal pouch interposition (JPI), double-flap technique (DFT), double-tract reconstruction (DTR), gastric tube reconstruction (GT), and side overlap with fundoplication by Yamashita (SOFY). Network meta-analyses were performed to consolidate outcome measures, such as reflux esophagitis (RE), postoperative reflux symptoms, and nutritional status.

**Results:**

This meta-analysis encompasses 16 studies published from 2009 to 2023, involving a total of 1,184 participants and examining 7 distinct reconstruction methods. The results indicated that patients who received DFT [odds ratio (OR) 13.27; 95% confidence interval (CI) 2.86–61.45] had a significantly lower rate of RE compared to those who underwent EG, as did patients who underwent SOFY (OR 4.58, 95% CI 1.16–18.10). Moreover, the DFT group demonstrated superior anti-reflux efficacy compared to the JI and DTR groups. Regarding nutritional status, both the JI group [standard mean difference (SMD) 12.16; 95% CI 3.57–20.76] and the DFT group (SMD 9.16; 95% CI 2.21–16.11) exhibited higher albumin levels compared to the EG interventions.

**Conclusion:**

DFT has shown promising efficacy in anti-reflux and postoperative nutritional status. Furthermore, SOFY demonstrates efficacy in the management of reflux, whereas JI is linked to improved postoperative nutritional outcomes following LPG. While providing valuable insights, it is important to note that the comparative analyses of JPI and SOFY rely on single-trial data with modest sample sizes, which may affect the precision of comparative effect estimates.

**Registration:**

This network meta-analysis was registered on the PROSPERO (CRD42023414346).

**Supplementary Information:**

The online version contains supplementary material available at 10.1007/s13304-025-02324-9.

## Introduction

Gastric cancer is a prevalent malignancy on a global scale and ranks as the fourth leading cause of mortality worldwide [1]. Over the decades, the pattern of GC in both of Western and Asian countries has changed, with an ascending proportion of GC located in the upper third of the stomach [2–4]. Total gastrectomy (TG) is the preferred surgical approach for proximal gastric cancer, as it ensures safety and comprehensive lymph-node dissection. However, malnutrition and weight loss are common complications after TG [5]. Multiple studies indicate that PG yields superior nutritional outcomes relative to TG, while preserving comparable oncological safety profiles [6–8]. Moreover, the sixth Japanese Gastric Cancer Guideline recommends PG as a viable option for upper third GC and adenocarcinoma of the esophagogastric junction, especially for cT1N0 [9].

Patients undergoing PG frequently experience intolerable reflux as a consequence of the removal of the cardia and the anti-reflux valve, which markedly diminishes their quality of life (Qol) [10]. To address this issue, several anastomoses have been proposed. In comparison to esophagogastrostomy, the novel anastomosis methods have been found to decrease the occurrence of postoperative reflux by widening the distance between the remnant stomach and esophagus [11, 12], reconstructing the cardia [13, 14], and other strategies, though the outcomes of studies are inconsistent and there is no consensus on the optimal anastomosis technique after PG. Understanding the characteristics of each surgical procedure is essential for selecting the appropriate reconstruction techniques, mitigating reflux, and minimizing postoperative complications.

To evaluate the reflux rates and nutritional outcomes associated with various anastomoses following laparoscopic proximal gastrectomy (LPG), we undertook a network meta-analysis (NMA). This analysis encompassed seven prevalent anastomotic techniques: esophagogastrostomy (EG), jejunal interposition (JI), jejunal pouch interposition (JPI), double-flap technique (DFT), double-tract reconstruction (DTR), gastric tube reconstruction (GT), and side overlap with fundoplication by Yamashita (SOFY). Our objective was to furnish evidence regarding the most effective anastomosis subsequent to LPG.

## Methods

This NMA was conducted in line with Preferred Reporting Items for Systematic Reviews and Meta-Analyses (PRISMA) [15] guidelines, which has been registered in the PROSPERO international database (CRD42023414346).

### Data sources and search strategy

A thorough investigation was carried out across five electronic databases (PubMed, Medline, Embase, Cochrane Library, and Web of Science) from their inception until March 1, 2023. The search utilized a combination of keywords and mesh terms [((((((((((esophagogastrostomy) OR ("double tract")) OR ("double flap")) OR ("Kamikawa")) OR ("valvuloplasty")) OR ("gastric tube")) OR ("jejunal interposition")) OR ("jejunal pouch interposition")) OR ("side overlap")) OR (SOFY)) AND ((((("proximal gastrectomy") OR ("laparoscopic proximal gastrectomy")) OR ("laparoscopic-assisted proximal gastrectomy")) OR ("proximal partial gastrectomy")) OR ("partial gastrectomy"))]. The objective of the search was to pinpoint pertinent studies concerning reconstruction methods for patients who have undergone LPG. Subsequently, references were manually screened for related literature, including editorials, reviews, meta-analyses, and the included studies, to retrieve additional relevant studies.

### Selection criteria

To qualify for the qualitative analysis, studies were required to adhere to the following criteria: (1) Participants: patients who underwent proximal gastrectomy for gastric cancer; (2) Surgical procedures: laparoscopic proximal gastrectomy or laparoscopic-assisted proximal gastrectomy; (3) Intervention: one of seven reconstruction methods after proximal gastrectomy, including esophagogastrostomy, double-tract reconstruction, jejunal interposition, gastric tube reconstruction, double-flap technique, jejunal pouch interposition, and side overlap esophagogastrostomy; (4) Outcome measures: primary outcomes include reflux esophagitis (RE) verified by endoscopy 12 months post-surgery, postoperative reflux symptoms at 6 months, and albumin levels at 6 months post-surgery. The secondary outcomes encompass complications occurring 6 months after surgery, the duration of postoperative hospitalization, and the operation time.

Exclusion criteria for this analysis were as follows: (1) Reviews, case reports, letters, conference abstracts, and non-comparative studies; (2) Studies involving total gastrectomy or reconstruction methods not among the seven strategies previously mentioned; (3) Studies using laparotomy operation; (4) Studies focused on basic science and animal studies; (5) Publications without sufficient data.

### Literature screening and data extraction

Two investigators (Chenglin Xin and Zimeng Wang) independently screened the full text in accordance with the inclusion and exclusion criteria. All discrepancies were addressed through dialog with a third investigator. Data were extracted from the included studies as follows: first author's family name, region and year of publication, study design, number of participants, and demographic characteristics of subjects.

### Assessment of risk of bias

Two evaluators independently performed a quality assessment for each included RCT trial using Review Manager 5.3 software. And the ROBINS-I ("Risk Of Bias In Non-randomized Studies of Interventions") tool [16] was used to evaluate the quality of the included controlled trials.

### Statistical analysis

The evaluations of continuous and binary variables were articulated through standardized mean difference (SMD) and odds ratio (OR), accompanied by the respective 95% confidence intervals (CI). The entire analysis was performed using the random effects model. The network meta-analyses were implemented using STATA (version 15.1) with the "mvmeta" command, along with the "network" and "network graph" packages.

Network data plots, funnel plots, and the surface under the cumulative ranking curve (SUCRA) were performed sequentially. The node-splitting method and loop-specific approach were adopted to evaluate the consistency between direct and indirect evidence. Finally, we created a comparison-adjusted funnel plot to investigate the possibility of publication bias. Contribution plot for the network is also calculated.

## Results

### Study characteristics and qualitative assessment

**Fig. 1:**
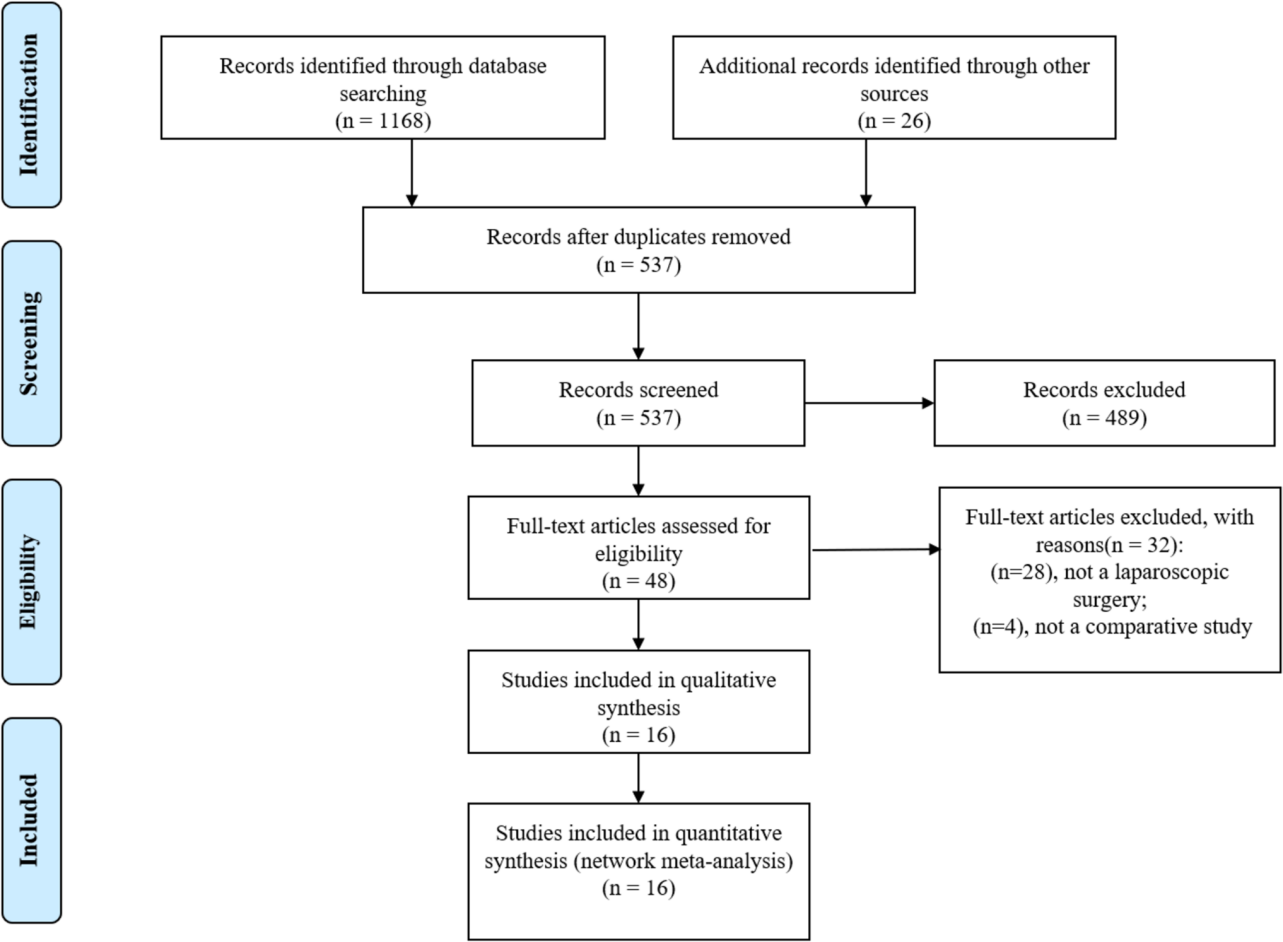
Flowchart of the literature screening, exclusion and inclusion process

In the course of the search process, a comprehensive screening of 1168 citations was conducted within electronic databases, complemented by the retrieval of additional 26 articles through the examination of related article references. Of these, 489 articles were excluded after reviewing article title and abstract against selection criteria. The full texts of the remaining studies were then carefully reviewed. For studies encompassing overlapping subjects (i.e., duplicates), only the report with the most comprehensive and informative data was included. Finally, 16 articles [14, 17–31] evaluating seven reconstruction methods, including EG, JI, JPI, DFT, DTR, GT, and SOFY, were selected for quantitative analysis. The study selection process is outlined in Fig. 1, highlighting the reasons for exclusion.

Table 1 delineates the fundamental characteristics of the chosen studies alongside the demographic details of the participants involved. A total of 1,184 participants across the seven reconstruction methods. DTR comprised 333 patients from 11 studies, EG comprised 263 patients across 11 studies, GT comprised 243 patients from 2 studies, DFT comprised 227 patients in 6 studies, and JI comprised 115 patients derived from 6 studies, while JPI and SOFY were each evaluated in single studies, involving 19 and 14 patients, respectively.

**Table 1:** Basic characteristics of the selected studies and demographic data for enrolled participants

Author	Year	Country	Reconstruction method	Sample size	Age (years)	Gender (M/F)	BMI (kg/m^2^)	Study design
Erica Nishimura et al. [17]	2023	Japan	EG	33	68 ± 4.7	22/11	22.1 ± 1.3	Retrospective
			DFT	35	67 ± 3.0	31/4	23 ± 1.1	Retrospective
			DTR	8	75.5 ± 3.8	8/0	22 ± 1.6	Retrospective
Bang Wool Eom et al. [18]	2021	Korea	EG	45	63.0 ± 4.7	33/12	25.0 ± 3.3	Retrospective
			DTR	58	65.5 ± 4.8	47/11	23.5 ± 3.1	Retrospective
Wataru Miyauchi et al. [19]	2020	Japan	EG	23	65 ± 14.0	15/8	23.0 ± 4.2	Retrospective
			DTR	24	73.5 ± 8.0	17/7	23.3 ± 4.05	Retrospective
Eiji Nomura et al. [20]	2014	Japan	DTR	10	65.8 ± 10.3	8/2	NM	Prospective
			JI	10	68.5 ± 6.2	7/3	NM	Prospective
Zenichiro Saze et al. [21]	2021	Japan	EG	9	74.33 ± 7.5	8/1	22.3 ± 2.9	Retrospective
			JI	10	71.5 ± 5.7	8/2	21.7 ± 2.3	Retrospective
			DTR	14	72.0 ± 10.0	12/2	22.5 ± 3.2	Retrospective
			DFT	36	70.1 ± 6.5	25/11	23.1 ± 3.1	Retrospective
Kei Hosoda et al. [22]	2019	Germany	EG	51	69.7 ± 9.0	38/13	23.1 ± 2.6	Retrospective
			DFT	40	69.5 ± 11.0	36/4	23.8 ± 3.0	Retrospective
Byunghyuk Yu et al. [23]	2022	Japan	DFT	18	63.2 ± 7.8	12/6	24.5 ± 3.5	Retrospective
			DTR	51	63.6 ± 11.4	38/13	23.5 ± 3.5	Retrospective
Tominaga et al. [24]	2021	Japan	EG	39	72 ± 10.2	28/11	22.2 ± 3.6	Retrospective
			DTR	17	68 ± 7.7	11/6	24.0 ± 3.1	Retrospective
Yoshitaka et al. [25]	2016	Japan	GT	84	64.6 ± 16.3	62/22	NM	Retrospective
			JI	40	66.0 ± 9.1	30/10	NM	Retrospective
Atsushi Yasuda et al. [26]	2015	Japan	EG	25	71.6 ± 8.6	18/7	NM	Retrospective
			JI	21	61.0 ± 9.5	13/8	NM	Retrospective
Eiji Nomura et al. [27]	2019	Japan	DTR	15	65.7 ± 10.6	13/2	NM	Prospective
			JI	15	69.3 ± 6.0	11/4	NM	Prospective
Shinichi et al. [28]	2009	Japan	DFT	26	65.3 ± 7.6	22/4	23.3 ± 3.1	Retrospective
			DTR	10	64.2 ± 7.2	8/2	22.2 ± 2.8	Retrospective
Tomoki Aburatani et al. [29]	2017	Japan	DTR	19	71 ± 9.9	16/3	21.4 ± 2.2	Retrospective
			EG	22	69 ± 10.2	17/5	22.0 ± 1.9	Retrospective
Ryo Takagawa et al. [30]	2010	Japan	JI	19	64.1 ± 6.75	16/3	23.5 ± 2.9	Retrospective
			JPI	19	66.5 ± 4.75	16/3	23.7 ± 3.2	Retrospective
Li Yang et al. [31]	2022	China	GT	159	64 ± 6.5	135/24	23.2 ± 3.6	Retrospective
			DFT	72	63 ± 5.5	57/15	23.4 ± 2.8	Retrospective
			DTR	107	62 ± 7.6	107/0	23.2 ± 3.6	Retrospective
Yoshito Yamashita et al. [14]	2016	Japan	SOFY	14	59 ± 10.5	11/3	22.8 ± 2.9	Retrospective
			EG	16	66 ± 10.5	11/5	21.8 ± 2.9	Retrospective

All eligible studies were published in English or Chinese between 2009 and 2023, with sample sizes ranged from 20 to 338. Two studies were prospective, while the rest were retrospective cohort studies. Each study was published as a full manuscript and was deemed to have a low risk of bias.

### Methodological quality assessment

**Fig. 2:**
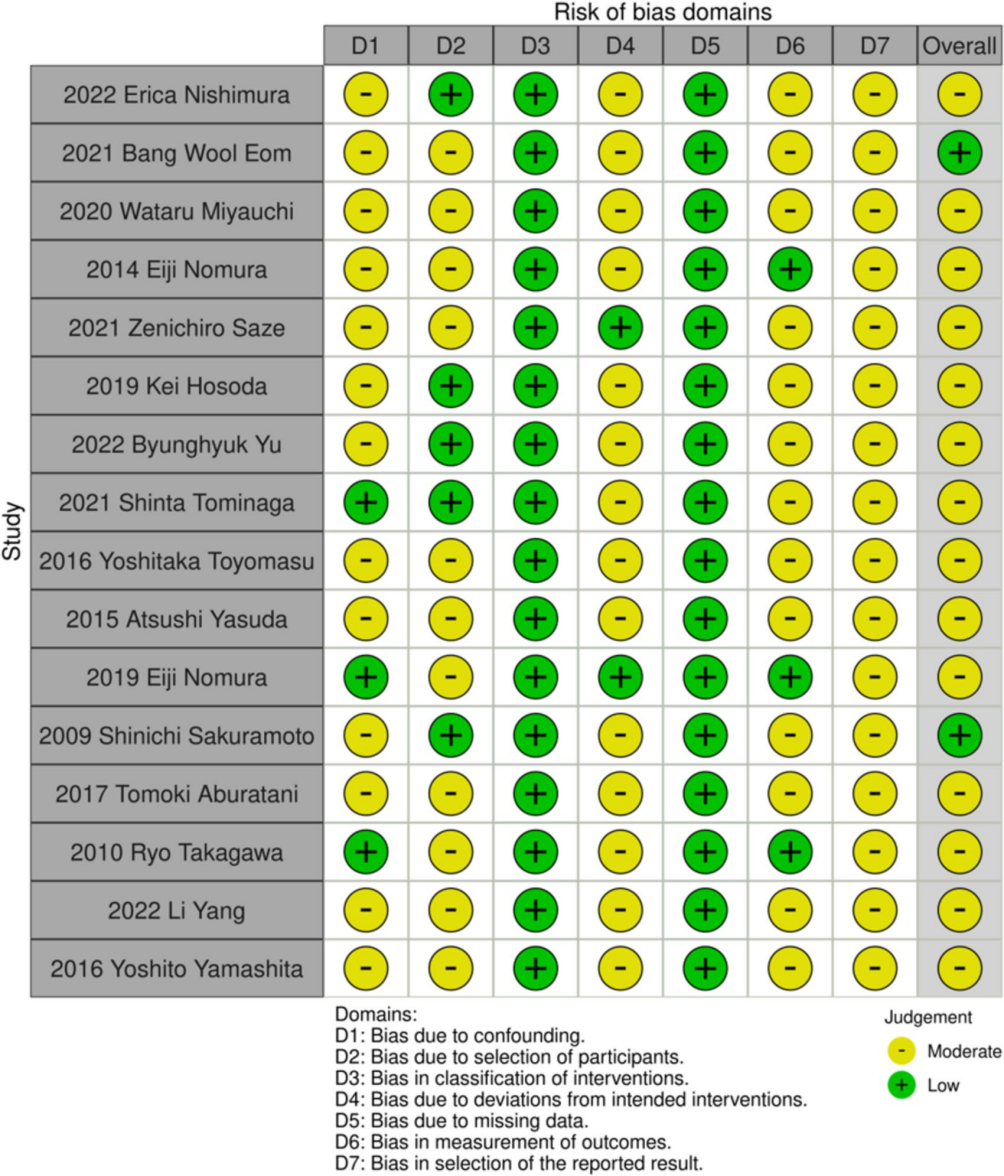
Risk-of-bias domains in selected studies

The studies' quality was assessed through the application of the ROBINS-I tool, which evaluates the risk of bias in non-randomized intervention studies. To accomplish our objective, we specified the research question, outcome, and result based on a target trial. We then assessed how confounders and co-interventions were managed for the specified outcome. Two researchers reached a consensus, as depicted in Fig. 2.

### Reflux esophagitis

**Fig. 3:**
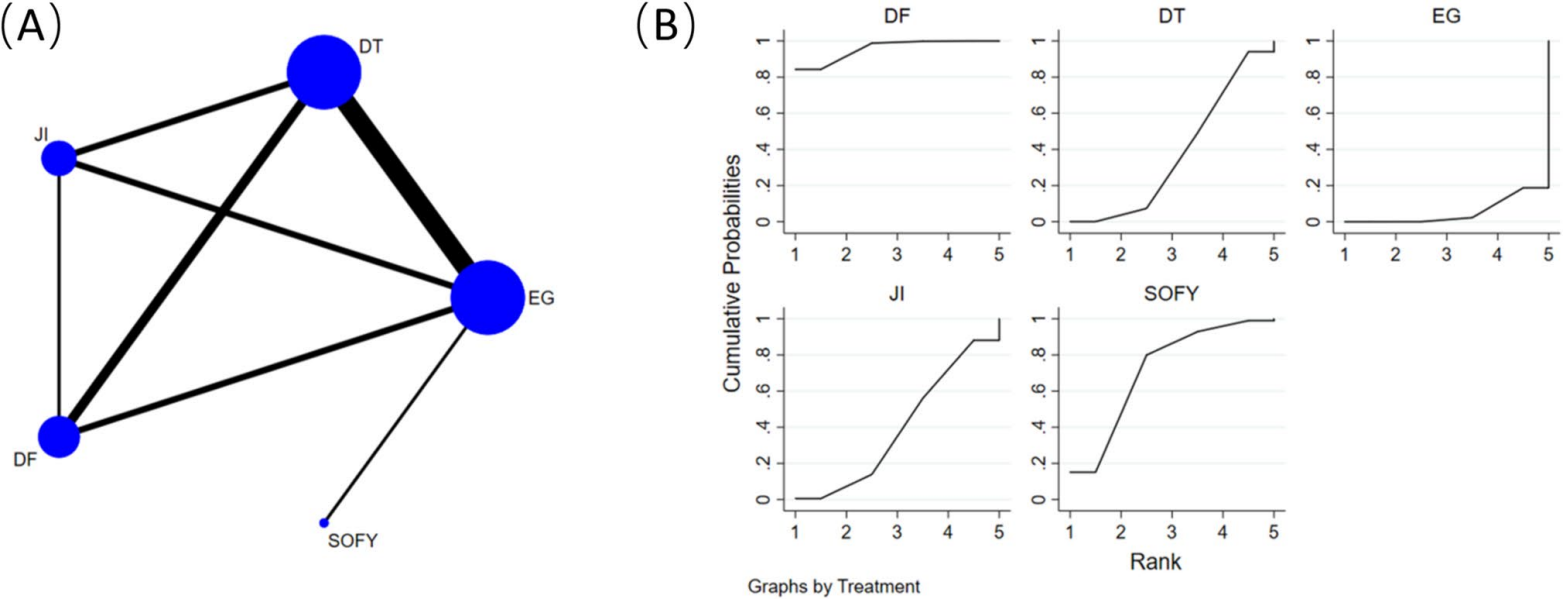
**A** Network plot of the comparisons for reflux esophagitis. **B** The cumulative ranking probability plot for each intervention across different reconstruction methods

Figure 3A shows ten retrospective cohort studies [14, 17, 18, 20, 21, 23, 24, 26, 28, 29], comprising data from 538 participants, including 5 reconstruction methods (EG, JI, DFT, DTR and SOFY) were reviewed to compare rates of RE. A network meta-analysis was carried out utilizing RE as the foundational framework for examination, with endoscopic verification executed for each method of reconstruction. Among the examined methodologies, DFT (OR = 0.08, 95% CI 0.02–0.35) and SOFY (OR = 0.22, 95% CI 0.06–0.86) exhibited reduced rates of postoperative RE in comparison to EG procedures. Additionally, DFT exhibited lower rates than JI and DTR procedures (Table 2).

**Table 2:** Network-comparison RE among five reconstruction methods

DFT				
0.35 (0.04,2.71)	SOFY			
0.14 (0.02,0.86)	0.42 (0.07,2.39)	JI		
0.13 (0.03,0.62)	0.39 (0.08,1.86)	0.93 (0.28,3.03)	DTR	
0.08 (0.02,0.35)	0.22 (0.06,0.86)	0.53 (0.18,1.55)	0.57 (0.26,1.21)	EG

Data shown as odds ratio with 95% confidence intervals (left versus right). *DFT* double-flap technique, *SOFY* side overlap with fundoplication by Yamashita, *JI* jejunal interposition, *DTR* double-tract reconstruction, *EG* esophagogastrostomy

Figure 3B presents the cumulative ranking probability plot and corresponding SUCRA values for each intervention across different reconstruction methods. The findings reveal that the DFT achieved the most elevated SUCRA value at 95.7%, while the SOFY followed with a value of 71.4%. In contrast, the DTR (42.3%), JI (39.9%), and EG (5.3%) had lower SUCRA values.

### Reflux symptom

**Fig. 4:**
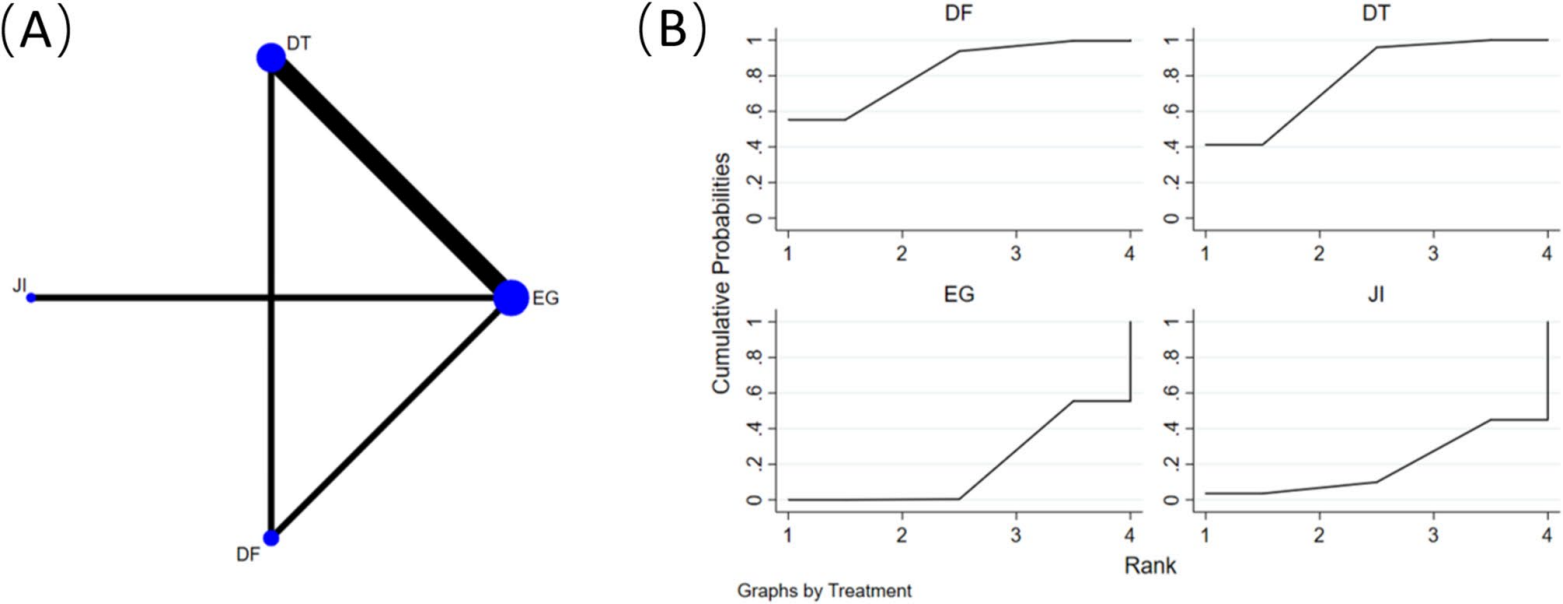
**A** Network plot of the comparisons for reflux symptom. **B** The cumulative ranking probability plot for each intervention across different reconstruction methods

Figure 4A shows that six retrospective cohort studies [19, 23, 24, 26, 28, 29], comprising data from 287 participants, including 4 reconstruction methods(EG, JI, DFT, and DTR) were reviewed to compare rates of reflux symptoms. An NMA was conducted utilizing reflux symptoms as the primary focus, with validation through questionnaires. The findings indicated that DFT (OR = 0.09, 95% CI 0.01–0.61) and DTR (OR = 0.11, 95% CI 0.04–0.35) interventions exhibited reduced frequencies of reflux symptoms compared to EG (Table 3).

**Table 3:** Network-comparison reflux symptom among four reconstruction methods

DFT			
0.84 (0.11,6.45)	DTR		
0.08 (0.00,2.42)	0.09 (0.00,2.04)	JI	
0.09 (0.01,0.61)	0.11 (0.04,0.35)	1.20 (0.07,21.27)	EG

Data shown as odds ratio with 95% confidence intervals (left versus right). *DFT* double-flap technique, *DTR* double-tract reconstruction, *JI* jejunal interposition, *EG* esophagogastrostomy

Figure 4B illustrates the cumulative ranking probability plot and corresponding SUCRA values for each intervention using different reconstruction methods. The results indicate that the DFT intervention achieved the highest SUCRA value (82.9%), followed by the DTR intervention (79.1%). In contrast, the JI and EG interventions had lower SUCRA values of 19.5% and 18.6% respectively.

### Albumin level six months after surgery

**Fig. 5:**
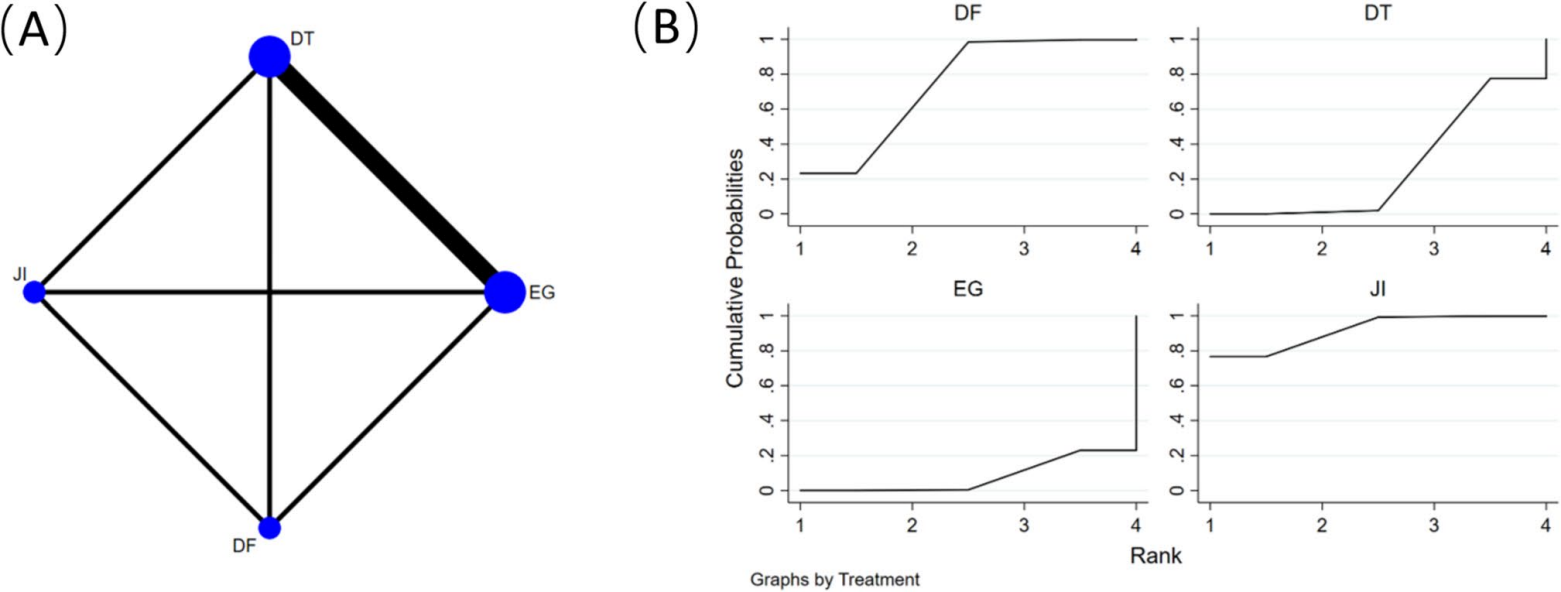
**A** Network plot of the comparisons for albumin level 6 months after surgery. **B** The cumulative ranking probability plot and for each intervention across different reconstruction methods

Figure 5A displays the comparison of albumin levels among four retrospective cohort studies [19, 21, 24, 29], involving 209 participants and utilizing 4 different reconstruction methods (EG, DFT, JI, and DTR). A network meta-analysis was performed utilizing albumin levels as the analytical foundation, indicating that JI (SMD = 12.16, 95% CI 3.57–20.76) and DFT (SMD = 9.16, 95% CI 2.21–16.11) interventions were linked to elevated albumin levels in comparison to EG interventions (Table 4).

**Table 4:** Network-comparison albumin level among four reconstruction methods

JI			
3.00 (-5.35,11.35)	DFT		
10.80 (2.28,19.32)	7.80 (0.95,14.65)	DTR	
12.16 (3.57,20.76)	9.16 (2.21,16.11)	1.36 (-2.14,4.86)	EG

Data shown as standard mean difference with 95% confidence intervals (left versus right). *JI* jejunal interposition, *DFT* double-flap technique, *DTR* double-tract reconstruction, *EG* esophagogastrostomy

Figure 5B displays the cumulative ranking probability plot and corresponding SUCRA values for each intervention using different reconstruction methods. The findings reveal that the JI intervention attained the most elevated SUCRA value at 91.9%, with the DFT intervention following at 73.8%. Conversely, the DTR (26.5%) and EG (7.8%) interventions exhibited diminished SUCRA values.

### Complication 6 months after surgery

**Fig. 6:**
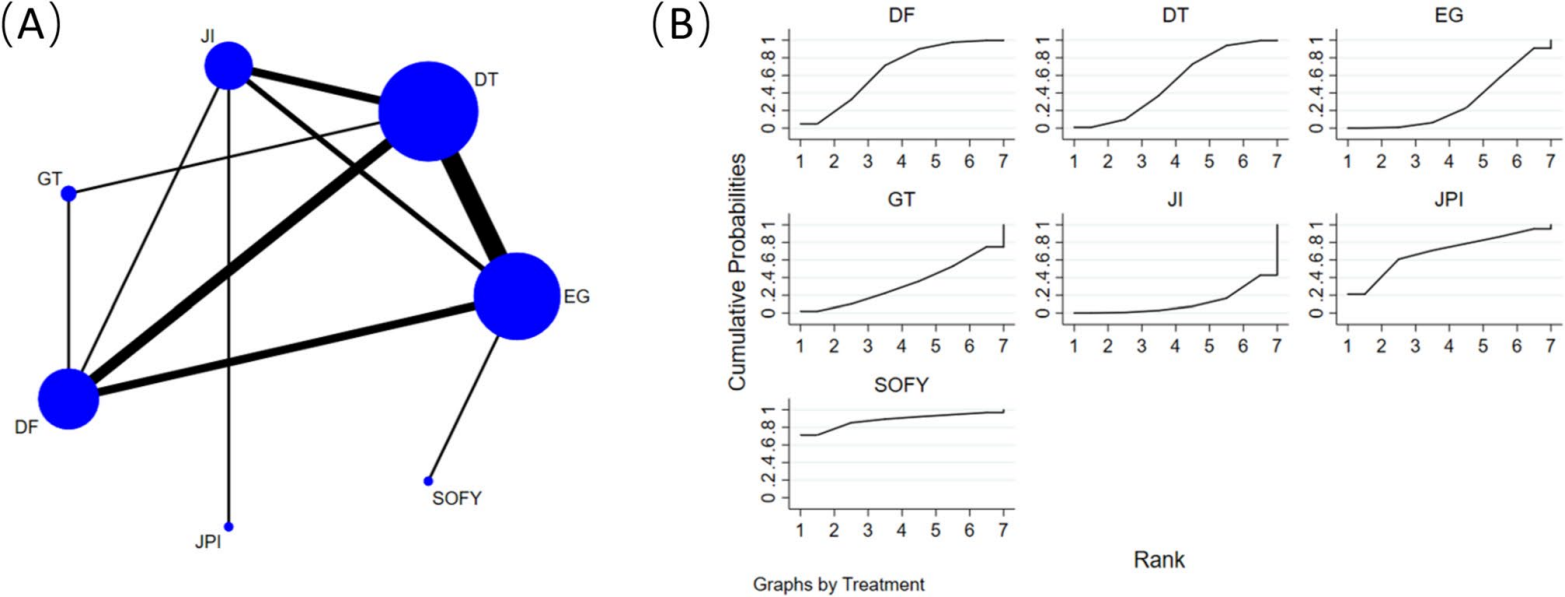
**A** Network plot of the comparisons for complication 6 months after surgery. **B** The cumulative ranking probability plot for each intervention across different reconstruction methods

Figure 6A illustrates the analysis of fifteen retrospective cohort studies [14, 17–24, 26–31], involving 1090 participants and comparing rates of postoperative complications among 7 reconstruction methods (EG, JI, JPI, DFT, DTR, GT, and SOFY). An NMA was conducted using postoperative complications as the basis of analysis, performed for each reconstruction method. No notable distinctions emerged among the various surgical interventions when evaluated through the lens of the consistency model (Table 5).

**Table 5:** Network-comparison complication among seven reconstruction methods

SOFY						
0.24 (0.00,18.15)	JPI					
0.15 (0.00,5.46)	0.64 (0.04,9.76)	DFT				
0.11 (0.00,3.78)	0.47 (0.03,6.45)	0.72 (0.28,1.84)	DTR			
0.07 (0.00,3.34)	0.30 (0.01,6.39)	0.46 (0.09,2.42)	0.63 (0.12,3.30)	GT		
0.07 (0.00,2.24)	0.30 (0.02,4.25)	0.47 (0.18,1.24)	0.65 (0.29,1.45)	1.02 (0.18,5.96)	EG	
0.04 (0.00,1.61)	0.17 (0.02,1.63)	0.26 (0.06,1.16)	0.36 (0.10,1.36)	0.57 (0.07,4.54)	0.56 (0.14,2.16)	JI

Data shown as odds ratio with 95% confidence intervals (left versus right). *SOFY* side overlap with fundoplication by Yamashita, *JPI* jejunal pouch interposition, *DFT* double-flap technique, *DTR* double-tract reconstruction, *GT* gastric tube reconstruction, *EG* esophagogastrostomy, *JI* jejunal interposition

Figure 6B presents the cumulative ranking probability plot and corresponding SUCRA values for each intervention across different reconstruction methods. The results show that SOFY intervention had the highest SUCRA value (88.2%), followed by JPI intervention (68.9%), and DFT (66.0%), DTR (52.3%), GT (33.0%), EG (29.9%), and JI (11.7%).

### Lengths of stay after surgery

**Fig. 7:**
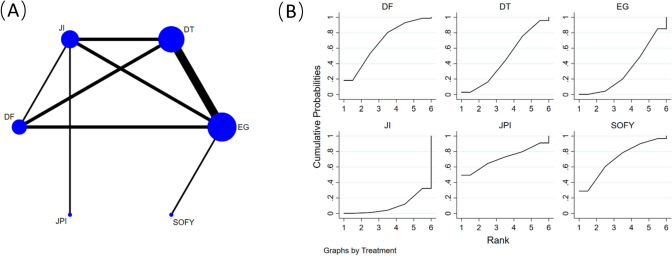
**A** Network plot of the comparisons for lengths of stay after surgery. **B** The cumulative ranking probability plot for each intervention across different reconstruction methods

Figure 7A shows that 11 retrospective cohort studies [14, 19, 21–24, 26–30], comprising data from 553 participants, including 6 reconstruction methods(EG, JI, JPI, DFT, DTR, and SOFY) were analyzed to compare lengths of stay (LOS) under different reconstruction methods. A network meta-analysis was performed utilizing the level of service as the foundational criterion for evaluation. No significant differences were found between surgical interventions when analyzed by the consistency model (Table 6).

**Table 6:** Network-comparison lengths of stay after surgery among six reconstruction methods

JPI					
− 1.03 (− 11.94,9.88)	SOFY				
− 1.52 (− 11.58,8.55)	− 0.49 (− 6.57,5.60)	DFT			
− 3.01 (− 12.74,6.72)	− 1.98 (− 7.66,3.70)	− 1.49 (− 5.34,2.36)	DTR		
− 3.87 (− 13.83,6.09)	− 2.84 (− 7.29,1.62)	− 2.35 (− 6.49,1.79)	− 0.86 (− 4.39,2.67)	EG	
− 5.90 (− 14.58,2.78)	− 4.87 (− 11.48,1.74)	− 4.38 (− 9.49,0.72)	− 2.89 (− 7.30,1.52)	− 2.03 (− 6.92,2.86)	JI

Data shown as standard mean difference with 95% confidence intervals (left versus right). *JPI* jejunal pouch interposition, *SOFY* side overlap with fundoplication by Yamashita, *DFT* double-flap technique, *DTR* double-tract reconstruction, *EG* esophagogastrostomy, *JI* jejunal interposition

Figure 7B illustrates the cumulative ranking probability plot along with the associated SUCRA values for each intervention across various reconstruction methodologies. The results show that JPI intervention had the highest SUCRA value (71.6%), followed closely by SOFY intervention (71.0%), and then DFT (68.7%), DTR (46.9%), EG (31.9%), and JI (10.0%).

### Operation time

**Fig. 8:**
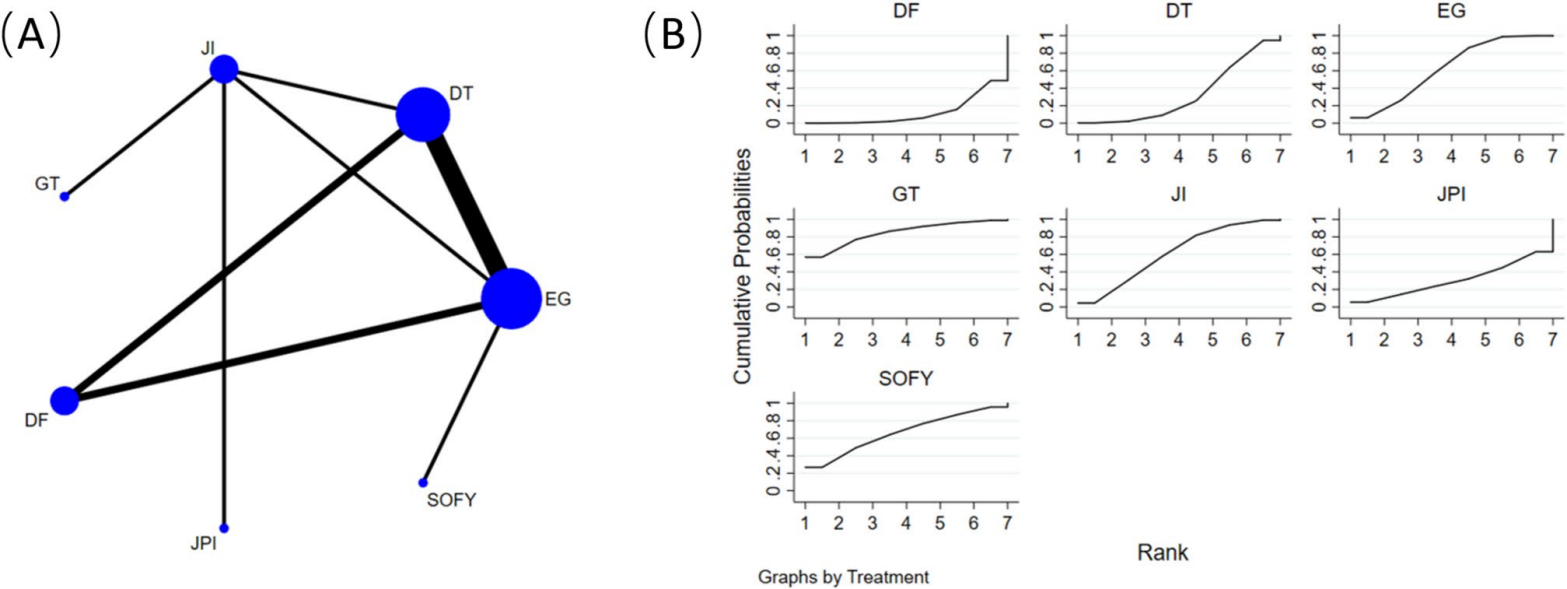
**A** Network plot of the comparisons for operation time. **B** The cumulative ranking probability plot for each intervention across different reconstruction methods

Figure 8A shows that 12 retrospective cohort studies [14, 17, 19, 22–30], comprising data from 684 participants, including 7 reconstruction methods (EG, JI, JPI, DFT, DTR, GT, and SOFY) were analyzed to compare operation times under different reconstruction methods. An NMA was conducted using operation time as the basis of analysis. The findings indicated that EG (SMD = -34.54, 95%CI: -67.88 to -1.19) correlated with reduced operation durations compared to DFT interventions; however, no notable differences emerged among the other surgical interventions when evaluated through the consistency model (Table 7).

**Table 7:** Network-comparison operation time among seven reconstruction methods

GT						
− 23.30 (− 73.28,26.69)	JI					
− 26.95 (− 93.08,39.18)	− 3.65 (− 46.96,39.65)	EG				
− 39.14 (− 116.79,38.52)	− 15.84 (− 75.27,43.59)	− 12.18 (− 52.88,28.52)	SOFY			
− 41.11 (− 107.41,25.20)	− 17.81 (− 61.38,25.76)	− 14.16 (− 40.84,12.53)	− 1.97 (− 50.69,46.75)	DTR		
− 50.30 (− 134.68,34.09)	− 27.00 (− 94.99,40.99)	− 23.34 (− 103.95,57.26)	− 11.16 (− 101.46,79.14)	− 9.19 (− 89.94,71.56)	JPI	
− 61.49 (− 133.43,10.45)	− 38.19 (− 89.94,13.55)	− 34.54 (− 67.88,− 1.19)	− 22.36 (− 74.83,30.11)	− 20.38 (− 54.89,14.13)	− 11.19 (− 96.63,74.24)	DFT

Data shown as standard mean difference with 95% confidence intervals (left versus right). *GT* gastric tube reconstruction, *JI* jejunal interposition, *EG* esophagogastrostomy, *SOFY* side overlap with fundoplication by Yamashita, *DTR* double-tract reconstruction, *JPI* jejunal pouch interposition, *DFT* double-flap technique

Figure 8B presents the cumulative ranking probability plot and corresponding SUCRA values for each intervention across different reconstruction methods. The results show that GT intervention had the highest SUCRA value (84.6%), followed by SOFY intervention (66.5%), and then EG (62.4%), JI (61.3%), DTR (32.5%), JPI (30.6%), and DFT (12.1%).

### Node-splitting method

The consistency of direct and indirect evidence was also assessed by node-splitting method. (Tables S1-6).

### Loop-closed inconsistency

Among the seven target outcomes, the evidence maps for each outcome encompassed loop closure, as illustrated in Fig. S1.

### Publication bias

As shown in Supplementary Fig. S2, all results reject the hypothesis of the existence of a small-study effect, as all plots were visually symmetric.

### Contribution plot

Contribution plot for the network is also calculated (Fig. S3). Consistency evaluation by the node-splitting method.

## Discussion

PG demonstrates a more favorable outcome in terms of postoperative nutrition and quality of life when contrasted with TG. Nonetheless, the issue of postoperative reflux continues to pose a considerable challenge for patients. A multitude of reconstruction methods has been put forth to tackle this issue, each possessing distinct characteristics. Despite this, there is a lack of comparative studies evaluating the anti-reflux effects and prognosis of these different methods. Therefore, a comparison of the efficacy of various reconstruction methods is necessary to inform clinical decision-making.

This systematic review meticulously examines 16 comparative studies involving 1184 participants diagnosed with PG, evaluating seven distinct reconstruction methods. Initially, our focus was on postoperative reflux, with the incidence of RE and the rate of reflux symptom. In the endoscopy examination conducted 12 months post-surgery, DFT exhibited lower rates of postoperative RE compared to the JI, DTR, and EG group. Consistent with previous research, our analysis underscores the substantial efficacy of the DFT in reducing postoperative RE and reflux symptoms [13, 32, 33], Hayami et al. reported 2.3% incidence of RE following DFT [32]. Moreover, a meta-analysis revealed that DFT exhibited a lower incidence of RE compared to JI, JPI, and EG, and was comparable to DTR [34].The procedure involves the wrapping of a double-muscle flaps at the anastomosis site between the esophagus and stomach, thereby creating a unidirectional valve. This method proficiently enhances pressure at the distal end of the esophagus, thereby aiding in the decrease of RE occurrence [13, 35]. Additionally, double-muscle flap anastomosis involves a single stoma surrounded by a seromuscular flap, leading to a lower incidence of anastomotic leakage [21].

SOFY has arisen as an alternative approach exhibiting favorable anti-reflux properties, by establishing an artificial gastric fundus that, when subjected to elevated pressure, generates a closed anastomosis, thereby effectively obstructing reflux [14]. In 2021, Yamashita et al. modified SOFY, demonstrating a postoperative endoscopic reflux esophagitis (RE) prevalence of 10.7% [36]. Prior research has demonstrated that mSOFY can significantly alleviate postoperative gastroesophageal reflux symptoms and eating discomfort, with efficacy and safety comparable to DTR [37]. However, the limited sample size in the study necessitates further research to validate its efficacy.

Regarding nutritional outcomes, the JI and DFT groups exhibited a superior nutritional status in comparison to individuals undergoing DTR and EG reconstruction. The superior performance of the JI technique in maintaining optimal postoperative nutritional status aligns with the findings of Lu et al. [38], who reported improved nutritional status in patients undergoing JI reconstruction compared to those undergoing DTR. The hypothesis suggests that a comprehensive gastroduodenal pathway for food intake could potentially improve the processes of digestion and absorption. Additionally, some studies have suggested that JI provides better feeding quality than EG [39], possibly due to its ability to function as a large remnant stomach substitute. Moreover, JI group exhibited higher body weight, hemoglobin, and vitamin B12 than the DTR group [38]. The increased intake of food through the residual gastric passage could be the reason for the heightened stimulation of the gastric cavity and the subsequent production of gastrin [27]. Gastrin is essential for the absorption of protein, iron, calcium, vitamin B12, and various other nutrients, as it facilitates the secretion of gastric acid [40].

The enhanced postoperative nutritional status observed in the DFT group can be attributed to a decrease in reflux incidents, resulting in improved food intake [32, 35]. Yu et al. proposed that the disparity between DFT and DTR may be attributed to variations in the amount of food passing through the remnant stomach and duodenum [23]. In patients with DFT, all consumed food is sent to the remaining stomach, while in DTR patients, around 60% of food avoids the remaining stomach [41]. Moreover, the delivery of food to the residual stomach is impeded after DTR, a phenomenon associated with adverse nutritional consequences [42]. Other studies have indicated that DFT may be more effective than JI in managing postoperative weight loss [43]. The difference in peristaltic movement between the esophagus and the interposed jejunum is thought to have potentially hindered the passage of food at the anastomosis.

Regarding operational duration, while DTR and JPI necessitate several anastomotic stomas, thereby augmenting the complexity of the procedure, recent advancements in laparoscopic technology and innovative anastomotic instruments have effectively reduced the disparities in operation time across different reconstruction techniques. However, the DFT method still necessitates manual anastomosis, leading to a significant prolongation of the operation time. Lately, using full laparoscopic anastomosis to shorten the duration of surgeries [44, 45].

The present study had several limitations. First, failure to report the baseline rate of RE and gastroesophageal reflux symptom, known to affect postoperative reflux-related outcomes in GC patients [46, 47]. Furthermore, future efforts should include enhanced endoscopic surveillance to assess long-term patterns of reflux progression, as well as the standardized application of LA classification criteria. This will enable stratified analysis of the efficacy of reflux control across different severity grades. Second, there are various reconstruction methods following PG, each with individual modifications. In our endeavor to encapsulate these procedures as delineated in the articles, it is important to acknowledge that technical variances may significantly influence research outcomes. Third, the predominance of retrospective cohort studies may introduce selection bias, highlighting the need for prospective research on postoperative quality of life, reflux status, nutritional outcomes, and complication rates across different proximal gastrectomy reconstruction methods.

Finally, JPI and SOFY were evaluated in single studies with small samples, leading to wider confidence intervals in their effect estimates. As highlighted by Caldwell et al. (48), such sparse data networks may lead to three specific biases. While we employed Bayesian priors to mitigate small-study effects, this approach cannot fully compensate for the absence of replication data(49). Clinicians should, therefore, regard conclusions regarding JPI and SOFY with caution.

## Conclusion

DFT has shown promising efficacy in anti-reflux and postoperative nutritional status. Furthermore, SOFY demonstrates efficacy in the management of reflux, whereas JI is linked to improved postoperative nutritional outcomes following LPG.

## Supplementary Information

Below is the link to the electronic supplementary material.

Supplementary file1 (DOCX 2302 KB)
