# Supplementary material for: Comparison on the reflux and nutritional status of different reconstruction methods after laparoscopic proximal gastrectomy: a systematic review and network meta-analysis
Source: Updates Surg. 2025 Nov 6;78(2):575–88. doi: 10.1007/s13304-025-02324-9 (PMC13212413; doi:10.1007/s13304-025-02324-9)
Supplement: Supplementary file 1 — Supplementary file1 (DOCX 2302 KB) [file 13304_2025_2324_MOESM1_ESM.docx]

**Comparison on the reflux and nutritional status of** **different reconstruction methods after laparoscopic proximal gastrectomy: a systematic review and** **network meta-analysis**


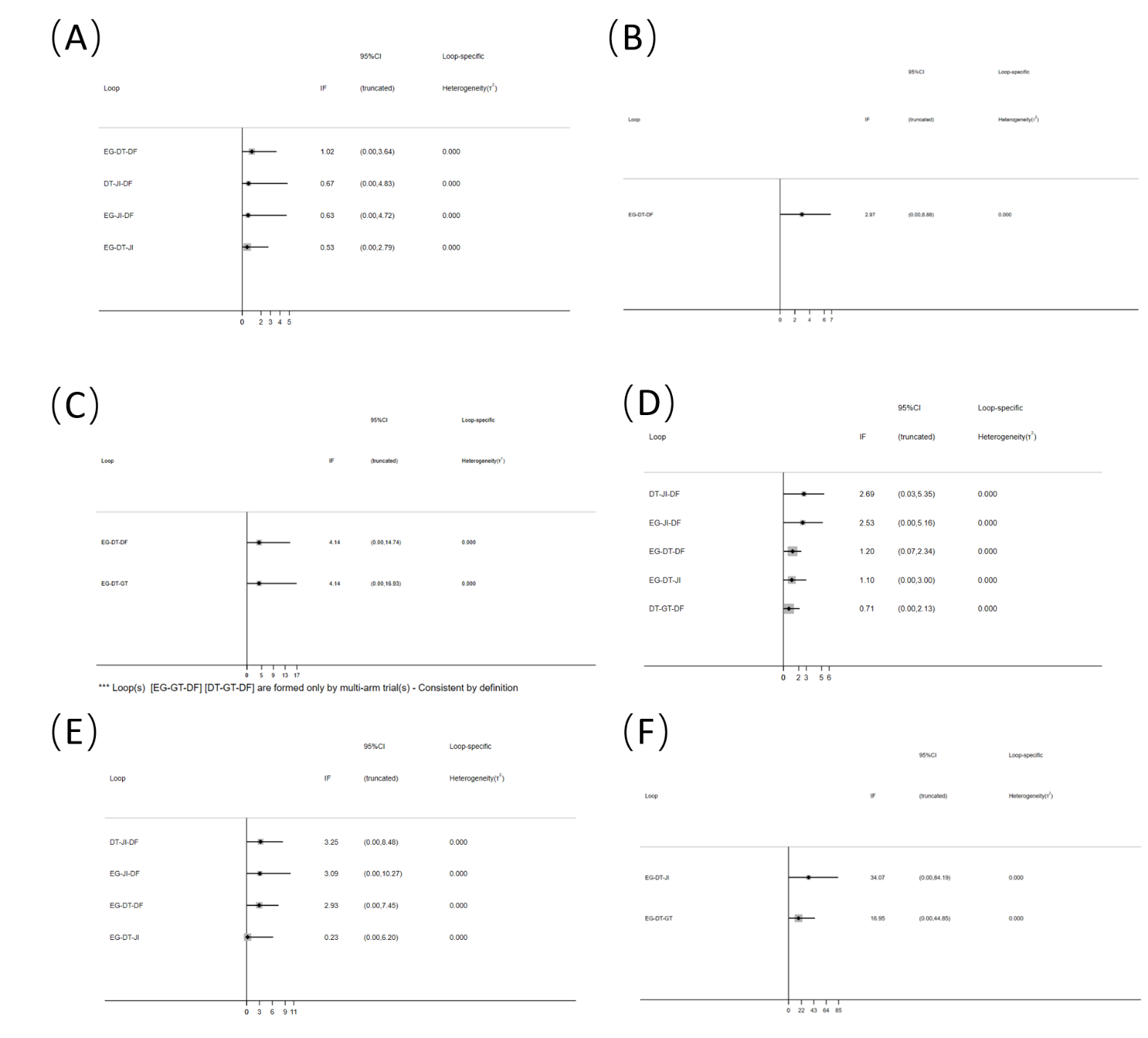


**Fig.S1** Loop-closed inconsistency (A) reflux esophagitis (B) reflux symptom (C) albumin level 6 months after surgery (D) complication 6 months after surgery (E) lengths of stay after surgery (F) operation time


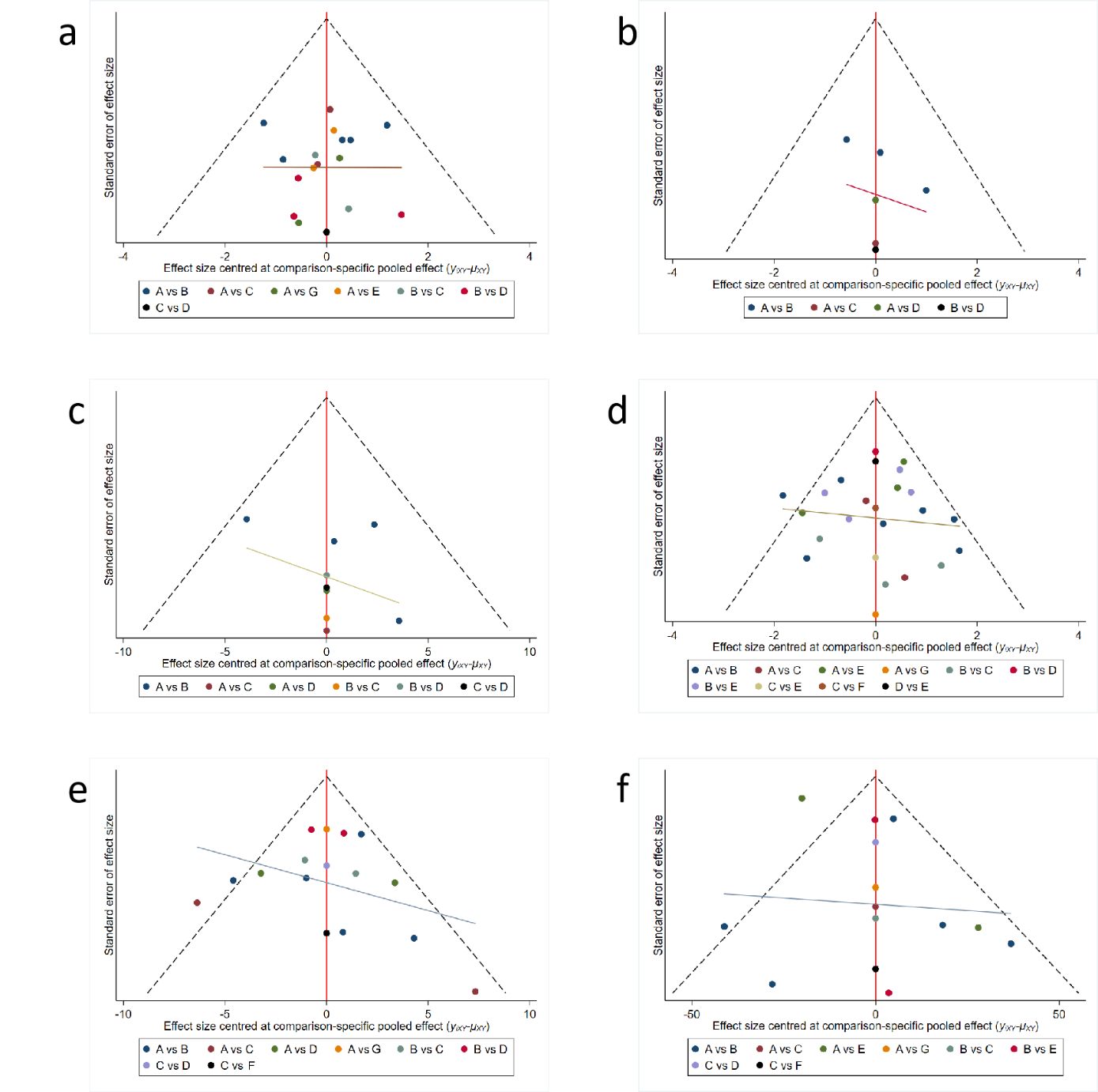
**Fig.S2** Publication bias (a) reflux esophagitis. (b) reflux symptom. (c) albumin level

six months after surgery. (d) complication 6 months after surgery. (e) lengths of stay after surgery. (f) operation time.


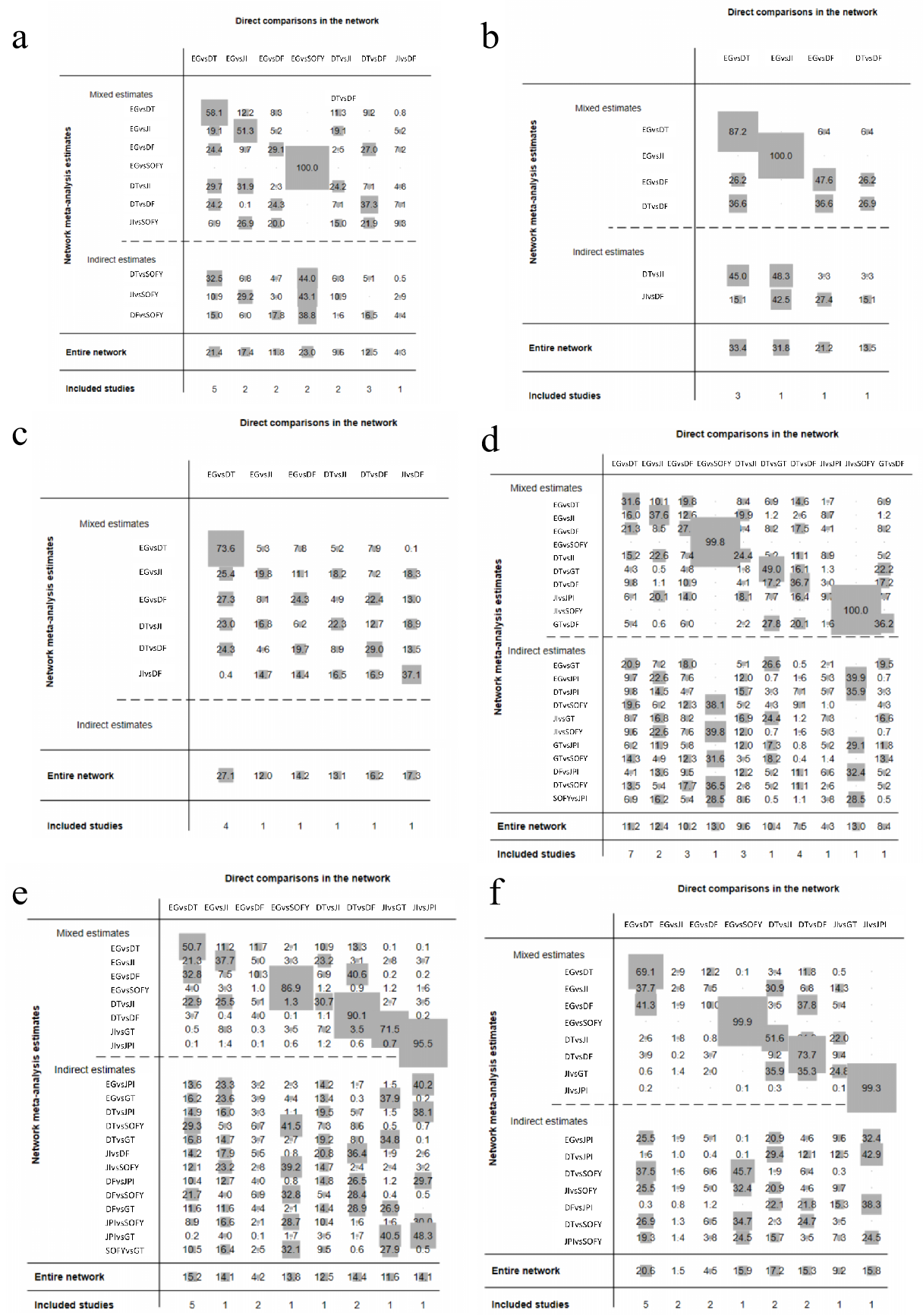


**Fig.S3** Contribution plot (a) reflux esophagitis. (b) reflux symptom. (c) albumin level 6 months after surgery. (d) complication 6 months after surgery. (e) lengths of stay after surgery. (f) operation time.

**Table S1** Consistency evaluation of reflux esophagitis by the node-splitting method.

| Side | Direct |  | Indirect |  |  | Difference |  | > tau |
| --- | --- | --- | --- | --- | --- | --- | --- | --- |
|  | Coef. | Std. Err. | Coef. | Std. Err. | Coef. | Std. Err. | P>\|z\| |  |
| EG DTR | -0.5519013 | 0.4180657 | -0.774989 | 1.420699 | 0.2230877 | 1.488268 | 0.881 | >0.195335 |
| EG DFT | -0.5603293 | 0.6125454 | -1.020276 | 1.263661 | 0.4599466 | 1.385381 | 0.740 | >0.1312581 |
| EG JI | -2.235045 | 0.8636051 | -3.4795 | 1.218915 | 1.244455 | 1.300944 | 0.339 | >2.08e-08 |
| EG SOFY | . | . | . | . | . | . | . | . |
| DTR DFT | -0.4861681 | 0.8640772 | 0.3056219 | 0.8323968 | -0.79179 | 1.17484 | 0.500 | >0.1203657 |
| DTR JI * | -2.396947 | 0.8140442 | 0.2116552 | 1.526118 | -2.608602 | 1.533812 | 0.089 | >4.57e-11 |
| DFT JI | -2.016908 | 1.587887 | -1.913152 | 1.096678 | -0.103756 | 1.883903 | 0.956 | >0.1366629 |

EG: esophagogastrostomy, DTR: double tract reconstruction, DFT: double flap technique, JI: jejunal interposition,SOFY: side overlap with fundoplication by Yamashita

**Table S2** Consistency evaluation of reflux symptom by the node-splitting method.

| Side | Direct |  | Indirect |  |  | Difference |  | >tau |
| --- | --- | --- | --- | --- | --- | --- | --- | --- |
|  | Coef. | Std. Err. | Coef. | Std. Err. | Coef. | Std. Err. | P>\|z\| |  |
| EG DTR | -2.39816 | 0.8085265 | 0.5033751 | 2.051084 | -2.901535 | 2.204697 | 0.188 | >0.5638974 |
| EG DFT | . | . | . | . | . | . | . | . |
| EG JI | -1.326871 | 1.296464 | -4.22822 | 1.783821 | 2.90135 | 2.205184 | 0.188 | >0.5640748 |
| DTR JI | -1.830448 | 1.58994 | 1.071331 | 1.527914 | -2.90178 | 2.20507 | 0.188 | >0.56404 |

| Side | Direct |  | Indirect |  |  | Difference | | >tau |
| --- | --- | --- | --- | --- | --- | --- | --- | --- |
|  | Coef. | Std. Err. | Coef. | Std. Err. | Coef. | Std. Err. | P>\|z\| |  |
| EG DTR | . | . | . | . | . | . | . | . |
| EG DFT * | 14.1 | 5.113227 | 5.962923 | 9.263486 | 8.137075 | 10.62682 | 0.444 | >2.20252 |
| EG JI * | 11.1 | 4.413488 | 2.962923 | 8.896402 | 8.137075 | 10.62682 | 0.444 | >2.20252 |
| DTR DFT * | 9.199997 | 4.888553 | 17.33707 | 9.620478 | -8.137075 | 10.62682 | 0.444 | >2.20252 |
| DTR JI * | 6.199997 | 4.151112 | 14.33707 | 9.267543 | -8.137075 | 10.62682 | 0.444 | >2.20252 |
| DFT JI | . | . | . | . | . | . | . | . |

**Table S3** Consistency evaluation of albumin level six months after surgery by the node-splitting method.

**Table S4** Consistency evaluation of complication 6 months after surgery by the node-splitting method.

| Side | Direct |  | Indirect |  |  | Difference |  | >tau |
| --- | --- | --- | --- | --- | --- | --- | --- | --- |
|  | Coef. | Std. Err. | Coef. | Std. Err. | Coef. | Std. Err. | P>\|z\| |  |
| EG DTR | -0.5577845 | 0.4660468 | 0.1268877 | 0.9663743 | -0.684672 | 1.072762 | 0.523 | >0.8977675 |
| EG DFT | 0.8755715 | 0.9310034 | 0.2168647 | 1.050043 | 0.6587068 | 1.374854 | 0.632 | >0.909071 |
| EG GT | -0.5923645 | 0.6394303 | -1.096233 | 0.9007829 | 0.5038687 | 1.110932 | 0.650 | >0.9127166 |
| EG SOFY | . | . | . | . | . | . | . | . |
| DTR DFT | 0.5279635 | 0.8145148 | 1.889464 | 1.061216 | -1.3615 | 1.289905 | 0.291 | >0.8272522 |
| DTR JI * | 0.5794879 | 1.01205 | -0.1291701 | 2.173001 | 0.708658 | 2.420662 | 0.770 | >0.9371804 |
| DTR GT * | -0.5689168 | 0.5360494 | 0.4938567 | 0.9967499 | -1.062773 | 1.138575 | 0.351 | >0.8372266 |
| DFT GT | -3.534291 | 1.178814 | -0.4338766 | 0.7618582 | -3.100415 | 1.348821 | 0.022 | >0.6176843 |
| DFT JPI * | -1.779337 | 1.157776 | -1.429869 | 133.3318 | -0.349468 | 133.3354 | 0.998 | >0.8564462 |
| JI GT * | -0.6415121 | 1.039791 | -1.350274 | 2.133385 | 0.7087624 | 2.420693 | 0.770 | >0.9371826 |

**Table S5** Consistency evaluation of lengths of stay after surgery by the node-splitting method.

| Side | Direct |  | Indirect |  |  | Difference | | >tau |
| --- | --- | --- | --- | --- | --- | --- | --- | --- |
|  | Coef. | Std. Err. | Coef. | Std. Err. | Coef. | Std. Err. | P>\|z\| |  |
| EG DTR | -1.753662 | 2.085147 | 2.678079 | 4.084735 | -4.431741 | 4.547217 | 0.330 | >3.197739 |
| EG DFT | 2.186358 | 3.427552 | 2.112399 | 4.11772 | 0.0739585 | 5.335441 | 0.989 | >3.321532 |
| EG JI | -4.212848 | 2.595284 | 1.613603 | 3.875649 | -5.826451 | 4.722268 | 0.217 | >3.027868 |
| EG SOFY | . | . | . | . | . | . | . | . |
| DTR DFT | 1.914421 | 2.622802 | 6.520278 | 4.844191 | -4.605857 | 5.431995 | 0.396 | >3.203727 |
| DTR JI | -0.952869 | 2.478253 | -3.11692 | 4.218276 | 2.164051 | 4.90877 | 0.659 | >3.312793 |
| DFT JI | -5.113913 | 3.760048 | -3.698504 | 4.163761 | -1.415409 | 5.611331 | 0.801 | >3.293143 |
| DFT JPI * | -5.9 | 4.427682 | -4.067124 | 545.5755 | -1.832876 | 545.6105 | 0.997 | >3.001495 |

**Table S6** Consistency evaluation of operation time by the node-splitting method.

| Side | Direct |  | Indirect |  |  | Difference | | >tau |
| --- | --- | --- | --- | --- | --- | --- | --- | --- |
|  | Coef. | Std. Err. | Coef. | Std. Err. | Coef. | Std. Err. | P>\|z\| |  |
| EG DTR | 11.87742 | 16.36862 | 23.27527 | 33.99217 | -11.39785 | 37.74925 | 0.763 | >26.9406 |
| EG DFT | -17.60001 | 30.66 | 14.39991 | 34.89825 | -31.99992 | 46.45346 | 0.491 | >25.5528 |
| EG GT | 43.32137 | 19.80308 | 2.849072 | 37.18978 | 40.47229 | 41.90688 | 0.334 | >24.53436 |
| EG SOFY | . | . | . | . | . | . | . | . |
| DTR DFT | -2.999995 | 31.52727 | -34.99704 | 34.12742 | 31.99705 | 46.46175 | 0.491 | >25.55351 |
| DTR GT | 3.693554 | 16.92582 | 63.29062 | 29.16714 | -59.59707 | 33.81414 | 0.078 | >18.69723 |
| DFT JI * | -23.3 | 25.50424 | 15.58566 | 3030.717 | -38.88566 | 3030.828 | 0.990 | >18.69723 |
| DFT JPI * | 27 | 34.68912 | 7.308018 | 4870.469 | 19.69198 | 4870.576 | 0.997 | >24.01246 |
